# Lotilaner - a novel formulation for cats provides systemic tick and flea control

**DOI:** 10.1186/s13071-018-2970-x

**Published:** 2018-07-13

**Authors:** Ian Wright

**Affiliations:** The Mount Veterinary Practice, 1 Harris Str., Fleetwood Lancs, FY7 6QX UK

Effective tick and flea control for pets is essential around the world for the health of companion animals, to limit zoonotic risk and to maintain a strong human-animal bond. Minimising pet exposure to ticks and preventing household infestations of fleas helps to prevent not only dermatitis and irritation but also vector-borne pathogen transmission to pets and people alike. A strong bond between owners and their pets has also been demonstrated to be beneficial to physical and psychological human health [1]. This bond is eroded by the presence of visible infestations and the feeling of revulsion they convey is reason enough for many owners to want to avoid infestation on their pets. Both the Companion Animal Parasite Council (capcvet.org) and the European Scientific Counsel Companion Animal Parasites (esccap.org) view tick and flea control as vital to maximise these benefits and reduce pathogen transmission. To achieve effective tick and flea control, highly efficacious adulticide products are required. Not only must these products eliminate ticks and fleas on the pet quickly, but they must also be easy to administer and affordable to the client, otherwise compliance will be lost. This is especially true in cats, where administration of treatment can be particularly challenging. Adverse behavioural and physiological reactions by cats to treatment administration can deter owners from treating them if other treatment modalities are then not available.

Just as the introduction of fipronil gave hope of safe flea control on dogs and cats, the development of modern tick and flea control products have brought increased flexibility and tick efficacy to the market. This is much needed at a time when flea challenge is increasing in most countries and climate change and increased movements of pets and people is fuelling increased distribution of ticks [2, 3]. The isoxazolines are highly efficacious novel ectoparasiticdes which form an important part of the arsenal that veterinary professionals have at their disposal to meet these challenges. Lotilaner, the novel isoxazline described in this article collection, provides a new tick and flea control option for cats which is not only highly efficacious, but also represents the first effective oral tick treatment product for cats.

This article collection contains a collection of papers evaluating the efficacy, safety and pharmaco-kinetics of lotilaner in cats. Elanco developed this drug as a highly efficacious and easy to administer solution to tick and flea infestations. The pharmacokinetic and safety studies described document that lotilaner is rapidly absorbed after oral administration and was not associated with any adverse side effects seen at rates above control groups, even at 5 times the maximum recommended dose [4]. The excellent oral safety profile of lotilaner is unique and especially important in cats, which are particularly susceptible to the toxic effects of certain ectoparasiticide drug classes such as pyrethroids and organophosphates [5].

The cat flea, *Ctenocephalides felis*, remains the predominant flea infesting cats worldwide and continues to infest homes, bringing misery to both pets and owners [2, 3]. Controlling infestations in as fast as possible time depends on a range of measures including effective treatment of the environment [3]. The most essential element in flea control, however, is treatment of all susceptible pets in a household frequently enough with an efficacious product to ensure flea egg production is halted. The experimental flea infestation studies in this article collection demonstrate that lotilaner begins killing fleas as as early as 8 hours post-administration and at 8 hours after subsequent weekly infestations of adult *C. felis* for at least 1 month [6]. The fast and sustained speed of kill of lotilaner is consistent with pharmacokinetic data in this article collection, showing that, following oral administration in fed cats, it is readily absorbed, with peak blood concentrations reached within 4 hours and a half-life of over 4 weeks [7]. Such rapid speed of kill not only results in breaking the flea life-cycle but also rapidly reduces flea biting which is beneficial in the reduction of flea allergic dermatitis (FAD). This was demonstrated in field trials, which confirmed an efficacy against fleas higher than 97% and that clinical signs of FAD were improved or eliminated [8].

The wide range of ticks infesting dogs capable of transmitting tick-borne disease, some of them zoonotic, has led to much attention from the media, veterinary professionals and industry. These ticks include *Ixodes* spp. capable of transmitting the agent of Lyme disease and tick-borne-encephaltitis, as well as *Rhipicephalus sanguineus* which not only transmits a wide range of pathogens, but can also infest homes and kennels [9]. What has received less attention, however, is that cats can and do also harbour these ticks. Cats are predominantly infested with *Ixodes* spp. [10, 11], but can also carry a wide range of other ticks such as *Dermacentor* spp. and *Rhipicephalus* spp. Although cats appear immunologically less susceptible to tick-borne disease and groom off many ticks before pathogen transmission can occur, there are tick-borne pathogens capable of causing potentially life-threatening disease in cats. These include *Cytauxzoon felis* in North America and *Babesia felis* in Africa [12, 13]. In addition, cats have the potential to bring ticks carrying pathogens of veterinary significance into closer proximity with domestic environments. “Babesia vulpes”, *Borrelia* spp. and *Anaplasma phagocytophylum* have all been found identified in *Ixodes* spp. found on cats [11]. The field studies in this article collection also found cats to be carrying *R. sanguineus* with the potential for cats to introduce this tick into homes where infestation might establish [14]. Effective tick treatments are therefore required to help mitigate these risks and the tick studies presented in this article collection demonstrate that lotilaner treatment readily eliminates infestation with *Ixodes ricinus* and ticks continued to be killed with high efficacy throughout the month following treatment [15]. Detailed studies with *I. ricinus* showed that lotilaner was effective against this tick within 12 h of treatment, reaching 100% efficacy within 24 h. It also sustained a rapid kill of newly infesting *I. ricinus* through 35 days [15]. The rapid onset of action and speed of kill are important to significantly reduce pathogen transmission. In an EU field study treating pet cats with lotilaner, *R. sanguineus*, *Ixodes hexagonus* and *Dermacentor reticulatus* were reduced by at least 96.4% as well as *I. ricinus* [14].

ESCCAP emphasises the importance of the availability of different methods of parasite preventive administration to maximise compliance, and in the field of cat tick prevention the lack of treatment options was a barrier for many years. Now a variety of treatment options are available but lotilaner represents the first efficacious oral option for the treatment of ticks and fleas on cats, addressing the needs of owners who would rather give their cat a tablet. This is only a useful alternative option for owners; however, if oral products are palatable and easy to administer. Studies in this article collection showed that lotilaner tablets were well accepted by cats [8]. Compliance remains vitally important in preventive medicine, for however efficacious and safe a product is, it will only be effective if administered correctly and consistently.

Pet ownership provides increased psychological and physical wellbeing and the human-animal bond is to be strongly encouraged. With pet ownership, however, comes the possibility of infestation with ticks and fleas and the health problems these may bring. The outdoor lifestyle and wildlife hunting activities that cats pursue place them in a position of introducing tick and flea vectors and the pathogens they carry directly into their own homes and other domestic situations. These risks can be mitigated, however, by vigilance and the application of effective parasiticides. Lotilaner is one such product, offering the novel option of both tick and flea treatment in an oral formulation. It provides safe and effective tick and flea control with rapid speed of kill and onset of action. In doing so, it provides veterinary professionals and cat owners another effective option in tick and flea control in the novel form of an oral formulation, in the ongoing battle to control ticks and fleas.

French translation of the Abstract is available in Additional file 1.

## Additional file

Additional file 1: French translation of the article. (PDF 58 kb)
