# Supplementary material for: Lotilaner - a novel formulation for cats provides systemic tick and flea control
Source: Parasit Vectors. 2018 Jul 13;11:407. doi: 10.1186/s13071-018-2970-x (PMC6043969; doi:10.1186/s13071-018-2970-x)
Supplement: Supplementary file 1 — French translation of the article. (PDF 58 kb) [file 13071_2018_2970_MOESM1_ESM.pdf]

Lotilaner - Une formulation innovante qui permet de lutter contre les tiques et les puces chez le chat

Ian Wright

The Mount Veterinary Practice, 1 Harris Str., Fleetwood Lancs FY7 6QX, Royaume-Uni

Correspondance : hammondia@hotmail.com

Lutter efficacement contre les tiques et les puces qui infestent les animaux de compagnie est essentiel pour protéger leur santé, réduire le risque de zoonoses et contribuer à entretenir un lien fort entre l'homme et l'animal. Limiter l'exposition aux tiques et empêcher les infestations domestiques par les puces contribue à prévenir les dermatites et les irritations mais aussi la transmission vectorielle d'agents pathogènes aux animaux de compagnie comme aux personnes. Il a également été démontré qu'un attachement fort entre un animal de compagnie et ses maîtres est bénéfique pour la santé physique et psychologique de l'homme [1]. Ce lien est affecté par la présence d'infestations visibles, et le sentiment de révulsion qu'elles suscitent suffit à justifier, pour beaucoup de propriétaires, la volonté d'éviter de telles infestations chez leurs animaux. Le CAPC (*Companion Animal Parasite Council* – capcvet.org) et l'ESCCAP (*European Scientific Counsel Companion Animal Parasites* – esccap.org) considèrent qu'il est primordial de lutter contre les tiques et les puces afin d'optimiser ces bénéfices et de réduire la transmission d'agents pathogènes. Le contrôle efficace des tiques et des puces exige l'emploi d'imagocides très puissants. En effet, ces produits doivent non seulement éliminer rapidement les tiques et les puces présentes sur l'animal, mais aussi être faciles à administrer et abordables pour le propriétaire, sans quoi l'observance du traitement sera insuffisante. Ceci est particulièrement vrai chez le chat, pour lequel l'administration d'un traitement peut s'avérer très difficile. En l'absence d'autres modalités thérapeutiques, les réactions indésirables observées chez le chat, tant sur le plan physiologique que comportemental, peuvent dissuader certains propriétaires de traiter leur animal.

Alors que la commercialisation du fipronil offrait l'espoir d'un contrôle sans danger des puces chez le chien et le chat, le développement de produits antiparasitaires modernes a apporté au marché plus de souplesse et d'efficacité dans la lutte contre les tiques. Une avancée très attendue à une époque où les puces constituent un problème grandissant dans la plupart des pays, et où le changement climatique et la mobilité accrue des animaux comme des personnes favorisent une plus large répartition des tiques [2, 3]. Les isoxazolines, nouveaux antiparasitaires

externes extrêmement efficaces, représentent une part importante de l’arsenal dont les vétérinaires disposent pour relever ces défis. Le lotilaner, isoxazoline innovante décrite dans cette série d’articles, offre une nouvelle option pour lutter contre les tiques et les puces chez le chat. Très efficace, il est aussi le premier traitement contre les tiques destiné aux chats à être administré par voie orale.

Cette série d’articles contient plusieurs publications évaluant l’efficacité, l’innocuité et la pharmacocinétique du lotilaner chez le chat. Ce médicament a été développé par Elanco comme une solution contre les infestations par les tiques et les puces extrêmement efficace et facile à administrer. Les études pharmacocinétiques et d’innocuité présentées indiquent que le lotilaner est rapidement absorbé après son administration par voie orale, et qu’il n’a été associé à aucun effet indésirable observé plus fréquemment que dans le groupe témoin, même à des doses 5 fois supérieures à la dose maximale recommandée [4]. Unique, l’excellent profil d’innocuité orale du lotilaner est particulièrement important chez le chat, qui est très sensible aux effets toxiques de certaines classes thérapeutiques d’antiparasitaires externes, comme les pyréthrinoïdes ou les organophosphorés [5].

La puce du chat, *Ctenocephalides felis*, reste la principale espèce de puces infestant les chats à travers le monde, et continue à se répandre dans les maisons, faisant le malheur des animaux et de leurs propriétaires [2, 3]. Pour contrôler les infestations le plus rapidement possible, il est nécessaire de mettre en œuvre un ensemble de mesures et notamment un traitement efficace de l’environnement [3]. Cependant, l'élément le plus fondamental de la lutte contre les puces consiste à traiter régulièrement tous les animaux à risque au sein du foyer à l’aide d’un produit efficace, afin d’interrompre la ponte. Les études d'infestations expérimentales par des puces citées dans cette série d’articles montrent que l’effet létal du lotilaner débute dès 8 h après administration et 8 h après les infestations hebdomadaires suivantes par des puces *C. felis* adultes, et ce pendant au moins 1 mois [6]. L’action insecticide rapide et persistante du lotilaner corrobore les données pharmacocinétiques présentées dans cette série d’articles, qui démontrent que, suite à son administration par voie orale chez des chats nourris, le principe actif est rapidement absorbé, avec des concentrations sanguines maximales atteintes en 4 heures et une demi-vie supérieure à 4 semaines [7]. Une telle rapidité d’action permet non seulement d'interrompre le cycle de vie des puces, mais aussi de limiter rapidement les piqûres de puces, ce qui constitue un véritable atout dans la réduction de la dermatite allergique aux piqûres de puces (DAPP). Ces effets ont été démontrés par des études de terrain qui ont confirmé une efficacité contre les puces supérieure à 97 %, ainsi qu’une amélioration ou une disparition des signes cliniques de DAPP [8].

La grande diversité d'espèces de tiques infestant les chiens et susceptibles de transmettre des maladies, y compris des zoonoses, a attiré l’attention des médias, des professionnels et de l’industrie de la santé vétérinaire. Parmi ces tiques, citons *Ixodes* spp., qui peut transmettre les agents étiologiques de la maladie de Lyme et de l’encéphalite à tiques, et *Rhipicephalus sanguineus*, capable non seulement d’inoculer de très nombreux pathogènes, mais aussi d’infester les maisons et chenils [9]. En revanche, le fait que les chats puissent aussi être porteurs de ces tiques est moins connu. Les chats sont principalement infestés par *Ixodes* spp. [10, 11], mais peuvent également héberger une large palette d’autres espèces comme *Dermacentor* spp. et *Rhipicephalus* spp. Les chats semblent moins sensibles d'un point de vue immunologique aux maladies transmises par les tiques. Ils éliminent un grand nombre d’entre elles lors de leur toilette avant la transmission des agents pathogènes. Il existe néanmoins des agents pathogènes transmis par les tiques susceptibles d’induire des maladies potentiellement mortelles chez le chat, parmi lesquels *Cytauxzoon felis* en Amérique du Nord et *Babesia felis* en Afrique [12, 13]. Les chats ont en outre la possibilité de ramener des tiques porteuses d’agents pathogènes d’importance vétérinaire à proximité plus immédiate des environnements domestiques. Les bactéries *Borrelia* spp. et *Anaplasma phagocytophylum,* ainsi que le protozoaire *Babesia vulpes* ont tous été isolés dans des tiques *Ixodes* spp. retrouvées sur des chats [11]. Les études de terrain mentionnées dans cette série d’articles ont également permis d’isoler *R. sanguineus* sur des chats susceptibles d’introduire cette tique dans les maisons qui pouvaient alors être infestées [14]. Des traitements antitiques efficaces sont donc nécessaires pour limiter ces risques. Les études présentées ici démontrent que le lotilaner élimine rapidement les infestations par *Ixodes ricinus* et que sa grande efficacité létale sur les tiques persiste pendant le mois qui suit le traitement [15]. Les études portant sur *I. ricinus* décrites ici ont démontré que le lotilaner est efficace contre cette tique dans les 12 h qui suivent le traitement, atteignant une efficacité de 100 % dans les 24 h. Son action létale rapide persiste également sur les nouvelles infestations d’*I. ricinus* pendant 35 jours [15]. La rapidité d’action et de l’activité insecticide sont des paramètres importants pour réduire de façon significative la transmission des agents pathogènes. Dans une étude de terrain menée dans l'UE sur des chats de compagnie traités au lotilaner, le nombre de *R. sanguineus,* d’*Ixodes hexagonus* et de *Dermacentor reticulatus* a été réduit d’au moins 96,4 %, tout comme celui d’*I. ricinus* [14].

L’ESCCAP souligne l’importance de la disponibilité de différentes méthodes d’administration préventive d’antiparasitaires afin d'optimiser l’observance. Concernant la prévention des tiques chez le chat, il affirme par ailleurs que l’insuffisance des options thérapeutiques a constitué un obstacle pendant de nombreuses années. Si diverses options sont aujourd’hui disponibles, le lotilaner représente la première formulation orale efficace dans le

traitement des tiques et des puces chez le chat, répondant ainsi au besoin des propriétaires qui préfèrent donner des comprimés. L'administration orale n'est une alternative pratique pour les propriétaires que dans la mesure où ces produits sont également savoureux et faciles d'utilisation. Les études décrites dans cette série d'articles ont montré que les comprimés à base de lotilaner sont très bien acceptés par les chats [8]. L'observance demeure essentielle en médecine préventive : en effet, aussi efficace et sûr qu'il soit, un produit ne sera efficace que s'il est administré correctement et de façon régulière.

Posséder un animal de compagnie augmente le bien-être psychologique et physique, et le lien homme-animal doit être vivement encouragé. Cependant, avoir un animal de compagnie implique un risque d'infestation par les tiques et les puces, et les problèmes sanitaires associés. Le mode de vie extérieur et les activités de chasse font que les chats sont susceptibles d'introduire les puces et les tiques, ainsi que les agents pathogènes dont elles sont porteuses, directement dans leur propre foyer et dans d'autres environnements domestiques. Ces risques peuvent cependant être limités grâce à une certaine vigilance et à l'application d'antiparasitaires efficaces. Le lotilaner fait partie de ces produits et offre une toute nouvelle option de traitement antipuces et antitiques par voie orale. Grâce à la mise en place rapide de la protection et à la vitesse de son activité insecticide, il permet de lutter contre les tiques et les puces efficacement et sans danger. Présenté sous une forme innovante à administration par voie orale, il offre ainsi aux vétérinaires comme aux propriétaires de chats une alternative supplémentaire dans leur lutte permanente contre les tiques et les puces.

Agrément éthique et autorisation de participation

Sans objet.

Autorisation de publication

Sans objet.

Disponibilité des données et supports

Sans objet.

Conflit d'intérêts

L'auteur n'a aucun conflit d'intérêts.

Financement

La publication de cet article est soutenue par Elanco.

Contributions des auteurs

L'auteur est l'unique contributeur.

Références bibliographiques

1. Barker SB, Wolen AR. The benefits of human-companion animal interaction: a review. J Vet Med Educ. 2008;35:487–95.
2. Coles TB, Dryden MW. Insecticide/acaricide resistance in fleas and ticks infesting dogs and cats. Parasit Vectors. 2014;7:8
3. Dryden MW. Flea and tick control in the 21st century: challenges and opportunities. Vet Dermatol. 2009;20:435–40.
4. Kuntz AE, Kammanadiminti S. Safety of lotilaner flavoured chewable tablets (Credelio™) after oral administration in cats. Parasit Vectors. 2018 (Série à paraître).
5. Linnett PJ. Permethrin toxicosis in cats. Aust Vet J. 2008;86:32–5
6. Cavalleri D, Murphy MG, Seewald W, Nanchen S. Laboratory evaluation of the efficacy and speed of kill of lotilaner (Credelio™) against *Ctenocephalides felis* on cats. Parasit Vectors. 2018 (Série à paraître).
7. Toutain CE, Seewald W, Jung M. Pharmacokinetics of lotilaner following a single oral or intravenous administration in cats. Parasit Vectors. 2018 (Série à paraître).
8. Cavalleri D, Murphy MG, Seewald W, Nanchen S. A randomised, controlled field study to assess the efficacy and safety of lotilaner (Credelio™) in controlling fleas in client-owned cats in Europe. Parasit Vectors. 2018 (Série à paraître).
9. Hansford KM, Pietzsch M, Cull M, Medlock JM. Brown dog tick infestation of a home in England. Vet Rec. 2015;176:129–30.
10. Claerebout E, Losson B, Cochez C, Casaert S, Dalemans A, De Cat A, et al. Ticks and associated pathogens collected from dogs and cats in Belgium. Parasit Vectors. 2013;6:183
11. Davies S, Abdullah S, Helps C, Tasker S, Newbury H, Wall R. Prevalence of ticks and tick-borne pathogens: *Babesia* and *Borrelia* species in ticks infesting cats of Great Britain. Vet Parasitol. 2017;244:129–35.
12. Rizzi TE, Reichard MV, Cohn LA, Birkenheuer AJ, Taylor JD, Meinkoth JH. Prevalence of *Cytauxzoon felis* infection in healthy cats from enzootic areas in Arkansas, Missouri, and Oklahoma. 2015. Parasit Vectors. 2015;8:13.

13. Penzhorn BL, Schoeman T, Jacobson LS. Feline babesiosis in South Africa: a review. Ann N Y Acad Sci. 2004;1026;183–6.
14. Cavalleri D, Murphy MG, Seewald W, Nanchen S. A randomised, controlled field study to assess the efficacy and safety of lotilaner (Credelio™) in controlling ticks in client-owned cats in Europe. Parasit Vectors. 2018 (Série à paraître).
15. Cavalleri D, Murphy M, Seewald W, Drake J, Nanchen S. Laboratory evaluation of the efficacy and speed of kill of lotilaner (Credelio™) against *Ixodes ricinus* ticks on cats. Parasit Vectors. 2018 (Série à paraître).
